# Epidemiology of human immunodeficiency virus-1 and hepatitis B virus co-infection and risk factors for acquiring these infections in the Fako division of Southwest Cameroon

**DOI:** 10.1186/s12889-015-2386-x

**Published:** 2015-10-17

**Authors:** Lauren Shevell, Henry Dilonga Meriki, Fidelis Cho-Ngwa, Crystal Fuller

**Affiliations:** Department of Epidemiology, Columbia University Mailman School of Public Health, New York, USA; Department of Microbiology and Parasitology, University of Buea, Buea, Cameroon; Department of Biochemistry and Microbiology, University of Buea, Buea, Cameroon

**Keywords:** HIV, Hepatitis B, Sexual risk factors

## Abstract

**Background:**

Past studies have demonstrated that a large population of Cameroonians are afflicted with human immunodeficiency virus (HIV) and/or hepatitis B virus (HBV) demonstrating a need for better prevention programs. We aim to describe the prevalence of HIV, HBV and HIV/HBV co-infection; examine the association between HIV and HBV; and determine risk correlates associated with HIV and HBV transmission in Southwest Cameroon.

**Methods:**

A cross-sectional, community-based surveillance study was conducted among adults in five hospitals , one in each of the five health districts of  the Fako division of the Southwest region of Cameroon. Participants underwent pre- and post-test counselling, a 30-question survey and blood draw for HIV and HBV serologic testing. To construct a final model, chi-squared tests and logistic regression were used to investigate associations.

**Results:**

Among 761 participants, 40.32 % were male, mean age was 35.21 ± 12.42 years, and the prevalence of HIV, HBV and HIV/HBV co-infection was 10.69 % , 9.86 % , and 1.16 % , respectively. There was no association between HIV and HBV infection. However, there was a statistically significant crude associated (*p*-value < 0.05) between HIV and three high-risk sexual behaviour variables: condom use, number of lifetime sexual partners, and age at first sexual intercourse. After adjustment, HIV status continued to be associated with number of lifetime sexual partners (adjusted odds ratio (AOR) = 2.26; 95 % confidence interval (CI) =1.22–4.17) and age at first sexual intercourse (AOR = 2.63; 95 % CI =1.44–4.81). In contrast, none of the high-risk sexual behaviours was associated with HBV.

**Conclusions:**

The prevalence of HIV and HBV was relatively high in the Southwest region of Cameroon, emphasizing the importance of intervention and treatment programs in this country. Additionally, the results from this study suggest that unlike HIV, HBV is not associated with sexual risk factors and may provide evidence that HBV is acquired through routes other than sexual transmission, warranting further investigation in this region.

## Background

Human immunodeficiency virus (HIV) and hepatitis B virus (HBV) are a causes of significant morbidity across the world [1, 2]. According to the World Health Organization, 35 million people around the world are living with HIV [1] and over 240 million individuals are infected with chronic HBV infection [2]. The global estimate of co-infection with both diseases is between 2–4 million individuals [3]. Sub-Saharan Africa takes the brunt of the HIV epidemic making up 68 % of the global HIV population, totalling 22.65 million people [4]. Sub-Saharan Africa also represents the second-largest epidemic of chronic HBV carriers after Asia, totalling 50 million individuals—around 15 % of global chronic carriers [5]. Studies on co-infection with HIV and HBV in sub-Saharan Africa are sparse, but recent studies in South Africa and Kenya found the prevalence of co-infection to be around 5 % and 6 %, respectively [6, 7]. In Cameroon, the prevalence of HIV is 4.5 % among adults [8] and the prevalence of chronic HBV is around 9 % [9, 10]. At the present, there is very little data on the prevalence of co-infection with HIV and HBV in the general population in Cameroon; however, a study amongst blood donors found the co-infection rate to be 1.8 % [10]. Thus, further research is needed to investigate the prevalence of HIV and HBV co-infection in Cameroon.

Past research has demonstrated that the prevalence of HBV is much higher among those who are HIV positive compared to those who are HIV negative. A study of AIDS patients being treated at the National Institute of Health in the United States found that individuals infected with HIV were around 10 times more likely to test positive for hepatitis B surface antigen (HBsAg) compared to non-infected individuals [11, 12]. Further evidence found that among HIV-positive American youth (ages 12–20) who were infected through sexual activity, HBV was twice as likely in HIV-positive females and seven times as likely in HIV-positive males compared to the base-rate of HBV infection in the United States [13].

These studies were done outside of sub-Saharan Africa where there is a low prevalence of HBV and HIV. In these areas, it is believed that individuals are infected with HIV and HBV around the same time and by similar routes of transmission that occur in adulthood, such as injection drug use and unprotected sexual intercourse [5, 14]. This differs from the route of transmission in sub-Saharan Africa, where evidence has demonstrated that HBV transmission is mainly horizontal among young children [14, 15]. HBV is easily transmitted through non-sexual routes and can be spread from person to person through a variety of means including contact between skin lesions, blood transfusion, sharing of instruments that may be contaminated with blood such as toothbrushes or razors, and even person-to-person bites [16]. Research supports the finding that most infected individuals in sub-Saharan African countries with a high prevalence of HBV are exposed to HBV by the age of five [5, 17]. Apart from perinatal transmission, HIV transmission in sub-Saharan Africa, on the other hand, usually takes place in adolescence or young adulthood at the commencement of sexual activity [5]. This demonstrates a different transmission pattern of infection with HIV and HBV among areas of high prevalence of the diseases. Studies looking at a possible association between HIV and HBV have been pursued in many sub-Saharan countries, with contradicting results.

In studies throughout sub-Saharan Africa, there has been strong evidence across different countries, diverse populations, sexes and ages that it is not associated with HBV in this part of the world. A study with participants from Uganda and Burundi found that HIV infection was strongly associated with sexually transmitted diseases (STDs), such as syphilis, but was not correlated with HBV [18], providing further evidence that HBV is likely spread through a different transmission route than sexual intercourse. A statistically significant association between HIV and HBV was also unapparent in a study comparing HIV-positive to HIV-negative pregnant women in Malawi [19]. An additional study in the Côte d’Ivoire, with a robust 429-person sample, also found no association between HBV and HIV among women visiting a gynaecological clinic [20]. A similar pattern was observed in a study of only male participants. A nested cohort of 559 Malawian men working at a sugar estate, reported the prevalence of HBV to be 16.9 and 14.4 % among those who were HIV-positive and HIV-negative, respectively, with a *p*-value of 0.46 [21]. In contrast, other studies have found the prevalence of HBV to be significantly higher among individuals who are HIV positive compared to those who are HIV negative. In Zimbabwe, a study of 282 individuals found a significant association between HIV antibodies and hepatitis B viral markers [22], indicating HIV is associated with HBV. An additional study at a hospital in Mulago, Uganda found 65.1 % of individuals with HIV sero-positivity compared to 41.9 % of HIV sero-negative individuals to test positive for hepatitis B core antibody. This association was highly statistically significant with a *p*-value = 0.0002 [23]. The diverging results from the presented studies calls for further investigation into the association between HIV and HBV.

It is well established that HIV is associated with high-risk sexual behaviour worldwide including in sub-Saharan Africa. A South African study found women with older partners and inconsistent condom use and men with genital ulcer disease to be at higher risk of HIV infection [24]. Furthermore, studies have found that HIV infected individuals in sub-Saharan Africa had a significantly higher number of sexual partners than uninfected individuals and that HIV infected males had sexual contact with female prostitutes significantly more often than uninfected males [25].

The purpose of this study was to provide measurement of the prevalence of HIV, HBV and HIV/HBV co-infection in Cameroon. It was important to gain a comprehensive understanding of the size of the infected population to allow for better allotment of resources in order to enhance prevention and treatment measures. Moreover, we attempted to gain a better understanding of the primary transmission route of each disease and, based on past studies, hypothesized that high-risk sexual behaviours are associated with HIV but not HBV. Additionally, we did not expect to find an association between and HIV and HBV in this population.

## Methods

### Sample

The study was made up of a cross-sectional convenience sample of 761 participants over the age of 18 who attended a free screening in one of five hospitals in the Fako division of Southwest Cameroon. Age under 18 was the only criteria excluding individuals from participation. Each hospital was located in one of the five health districts of the Fako division: Muea (Muea Health centre), Tiko (Tiko Central clinic), Limbe (Limbe Regional Hospital), Muyuka (Muyuka District Hospital) and Buea (Buea Regional Hospital). The study was conducted from June 13th to July 6th, 2011. During this period, data was collected for 5 days from each of the five hospitals. Between 150 and 250 participants took part in the study in each hospital.

### Recruitment

Participants were recruited through many means including television, radio, community informers, and informative posters and pamphlets. We advertised free testing for HIV and HBV on the local television channel in the area and over the radio 3 days prior to and during the entire study. Recruitment posters were mounted throughout the towns and around the health centres where the screenings took place. Information about the study was also spread by word over a megaphone by town criers who dispensed important information to the community. Furthermore, announcements were made and flyers handed out at busy marketplaces.

### Screening

Upon arrival at the screening at one of the designated health centres, subjects took part in pre-test counselling with an experienced HIV counsellor. Each of the counsellors was recruited from the hospital where the screening was taking place and worked at the hospital as an HIV counsellor. The counsellor educated the participant on transmission risk factors and means to avoid contraction of HIV and HBV. The counsellor then administered a 30-question survey, detailed below, and the patient signed the IRB-approved consent form. After completing these steps, the participant had 2 mL of blood drawn by a qualified laboratory technician. The technician then ran the blood samples using rapid HIV and HBV test kits and results were available for the patient about 60 minutes later. The laboratory technicians were recruited from the hospital where the screening took place. Upon receiving results, patients underwent post-test counselling, which was dependent on the test outcome. HIV-positive patients were directed towards HIV treatment centres in the area to receive appropriate follow-up care. Those who were HBV-positive were told to consult a physician for proper care and further viral load testing. Those who were HIV and HBV negative were given further instructions on means to protect themselves from HIV and HBV infection in the future.

### Consent

This study was approved by the Cameroon National Ethics Committee (CNEC) and administrative authorisations were obtained from the Southwest Regional Delegation for Public Health and the health institutions where participants were enrolled. Participants were required to sign a consent form outlining the study procedures and detailing the risks involved in the study. For participants who were unable to read or understand the consent, the counsellor verbally explained the information.

### Measures

#### Questionnaire

The counsellors administered a questionnaire to each participant that included questions on demographics, past HIV and HBV testing practices, sexual practices and drug use. Questions on demographics included age, sex, marital status, pregnancy status, religion, education, career, and income. Questions about HIV and HBV testing included the number of times the participant had been tested for each disease, the reason for being tested, the results of the test, and if positive, age of diagnosis and whether the patient was receiving ART if HIV positive. Questions pertaining to sexual practice included age of first intercourse, number of sexual partners in the last year and lifetime, history of sexually transmitted diseases, frequency of condom use and whether the participant had sexual intercourse with a known HIV-positive person. Finally, the questionnaire contained additional questions on known risk factors for HIV and HBV acquisition such as injection drug use and blood transfusions.

#### Disease measurement

Three test kits were used in this study. The Determine HIV-1/2 test kit, which tests for the presence of HIV-antibodies in serum was used to screen all participants for HIV. Those who tested positive on the Determine test kits were confirmed using the HEXAGON HIV 3rd Generation Immuno-chromatographic Rapid Test for the Detection of Antibodies to Human Immune Deficiency Viruses 1 and 2. Hepatitis B virus was diagnosed by screening for the hepatitis B surface antigen (Acon Laboratory) using the DiaSpot HBsAg: One Step Hepatitis B Surface Antigen Test Strip. Each of these tests kits had sensitivity >99 % and a specificity >97 %.

#### Socio-demographic variables

The following socio-demographic variables were included in the analysis: age, sex, education level, religion, marital status, income level, and blood transfusion status. The age variable was divided into older and younger age, depending on the median age of 32. Sex was coded as either male or female. Education level was a binary variable compromised of low education (completion of no school or primary school) and high education (completion of secondary school or post-secondary education). Four religion variables were examined: Catholic and not Catholic, Protestant and not Protestant, Muslim and not Muslim, and other and not other. Marital status was divided into a binary variable made up as ever married (married, separated/divorced, and widowed) and never married (single). Income, which was divided into four levels in the questionnaire (10,000 CFA, 10–50,000 CFA, 50–100,000 CFA and >100,000 CFA), was a binary variable in the model divided into high (>50,000 CFA/month) and low (≤50,000 CFA/month), based on the median income level of 10,000–50,000 CFA per month. Students and housewives were excluded from income level analysis as they had incomes of zero, which was not necessarily representative of their socioeconomic statuses. Blood transfusion status was divided into simply “yes” to indicate ever having a transfusion and “no” to mean never having a transfusion.

#### Sexual behaviour variables

Sexual risk factors examined in the analysis included condom use, lifetime number of sexual partners, and age at first sexual intercourse. Condom use was a binary variable divided into low (never, rarely, and sometimes) and high (often, usually). Lifetime number of sexual partners was a binary variable divided into 0–9 and 10+. Age at first sex was a binary variable divided into less than 18 years old and greater than 18 years old, based on the median value of age at first sexual intercourse, 18.

### Statistical analysis

The data was analysed using SAS 9.2 looking for the prevalence of HIV, HBV, HIV/HBV co-infection and a potential association between HIV and HBV. Additionally, we looked at whether high-risk sexual behaviour variables were associated with each disease. To conduct final model building, chi-squared tests and logistic regression were used to investigate associations.

### Model building

Chi-squared analysis was used to analyse the crude relationship between each of the above variables in the questionnaire and HIV status and HBV status. Multivariate logistic regression models were built based on the results of the Chi-squared test and univariate logistic regression; only significant variables (*p*-value <0.05) were included in the final model.

## Results

### Study population description

The ages in the sample ranged from 18 (due to the bounds of the ethical clearance) to 94, with a mean age of 35.21  ± 12.42 years and a median age of 32. The sample was split fairly evenly in terms of gender, with 40.32 % of the sample made up of males and 59.68 % females. The study participants represented diverse religious backgrounds with 36.72 % Catholic, 39.51 % Protestant, 0.92 % Muslim and 22.85 % others. In terms of marital status, 51.71 % of the sample was single, 38.29 % was married, 2.76 % was divorced or separated and 7.24 % was widowed. Levels of education varied with 26.85 % of the sample completing some primary school, 31.12 % completing secondary school, 39.10 % completing post-secondary (university) education and 2.93 % having no school (Table 1).

**Table 1:** Socio-demographic characteristics of 761 adults in the Fako division of Southwest Cameroon (2011)

Variable	Category	Number	Percent
HIV	Negative	677	89.31
	Positive	81	10.69
HBV	Negative	686	90.14
	Positive	75	9.86
Sex	Male	306	40.32
	Female	453	59.68
Age	≤32	401	52.69
	>32	360	47.31
Marrital Status	Ever Married	367	48.29
	Never Married	393	51.71
Religion			
Catholic		278	36.72
Protestant		299	39.50
Muslim		7	0.92
Other		173	22.85
Education	Low	224	29.79
	High	528	70.21
Income	Low	367	62.63
	High	219	37.37
Blood transfusion	No	701	92.36
	Yes	58	7.64
Condom use	High	135	18.75
	Low	585	81.25
Lifetime sexual partners	<10	648	85.94
	≥10	106	14.06
Age at first sex	>18	295	38.76
	≤18	466	61.24

### Disease prevalence

The overall prevalence of disease in the sample population was 10.69, 9.86 and 1.18 % for HIV, HBV and HIV/HBV co-infection, respectively. There was not a significant association between HIV and HBV status (OR = 1.16, 95 % CI = 0.55–2.42).

### Factors associated with disease prevalence

In a univariate analyses, HIV status was associated with age, marital status, education level, condom use, lifetime number of sexual partners and age at first sexual intercourse. Older age (over 32) was associated with increased risk of HIV (OR = 2.95, 95 % CI = 1.79–4.86), never being married was protective from HIV compared to ever being married (OR = 0.58, 95 % CI = 0.36–0.92). High education level was protective from HIV compared to low education level (OR = 0.31, 95 % CI = 0.19–0.50), low condom use was associated with increased risk of HIV compared to high condom use (OR = 2.20, 95 % CI = 1.03–4.68). Similarly, more than 10 sexual partners was associated with increased risk of HIV compared to fewer than 10 sexual partners (OR = 2.69, 95 % CI = 1.56–4.62), and younger age at first sexual intercourse was also associated with increased risk for HIV compared to older age of first sexual intercourse (OR = 2.07, 95 % CI = 1.22–3.52) (Table 2).

**Table 2:** Demographic and sexual risk behaviour variables associated with HIV among adults in the Fako division of Southwest Cameroon (2011)

Variable	HIV positive *n* = 81	Crude OR (95 % CI)	Adjusted OR (95 % CI)
HBV test results			
Negative	72	1.16 (0.55–2.42)	1.68 (0.73–3.85)
Positive	9		
*Socio-demographics*			
Sex			
Male	26	1.45 (0.89–2.37)	-
Female	54		
Age			
≤ 32	24	2.95 (1.79–4.86)***	2.35 (1.24–4.46)**
> 32	57		
Marital status			
Ever married	49	0.58* (0.36–0.92)	1.09 (0.59–2.00)
Never married	32		
Religion			
Catholic			
No	52	0.92 (0.57–1.49)	-
Yes	28		
Protestant			
No	50	0.92 (0.57–1.48)	-
Yes	30		
Muslim			
No	79	1.41(0.17–11.86)	-
Yes	1		
Others			
No	59	1.22 (0.72–2.08)	-
Yes	21		
Education			
Low	44	0.31 (0.19–0.50)***	0.39 (0.22–0.69)**
High	37		
Income			
Low	59	0.45 (0.25–0.79)**	0.54 (0.28–1.02)
High	17		
Blood transfusion			
No	72	1.60 (0.75–3.39)	-
Yes	9		
*Sexual risk factors*			
Condom use			
High	8	2.20 (1.03–4.68)*	0.99 (0.42–2.30)
Low	72		
Lifetime sexual partners			
< 10	58	2.69 (1.56–4.62)**	2.26 (1.22–4.17)**
≥ 10	22		
Age at first sex			
> 18	20	2.07 (1.22–3.52)**	2.63 (1.44–4.81)**
≤ 18	61		

*CI* confidence interval; *OR* odd ratio

Please note that all reference categories are listed as the first category

Although the total sample is 81, one data point is missing from religion, sex, income level, condom use, and lifetime sexual partners. 14 data points were omitted from income level because the participant was a student or housewife

*indicates a *p*-value <0.05, **indicates a *p*-value <0.01,*** indicates a *p*-value < 0.0001

HBV was only associated with marital status and sex. Never being married increased the risk of HBV compared to ever being married (OR = 1.65, 95 % CI = 1.01–2.69) and females were at decreased risk compared to males for acquiring HBV (OR = 0.47, 95 % CI = 0.29–0.75) (Table 3).

**Table 3:** Demographic and sexual risk behaviour variables associated with HBV among adults in the Fako division of Southwest Cameroon (2011)

Variable	HBV positive *n* = 75	Crude OR (95 % CI)	Adjusted OR (95 % CI)
HIV			
Negative	66	1.16 (0.55–2.42)	1.36 (0.64–2.88)
Positive	9		
*Socio-demographics*			
Sex			
Male	43	0.47 (0.29–0.75)**	0.46 (0.28–0.75)**
Female	32		
Age			
≤ 32	45	0.72 (0.44–1.17)	
> 32	30		
Marital status			
Ever married	28	1.65 (1.01–2.69)*	1.66 (1.01–2.73)*
Never married	47		
Religion			
Catholic			
No	43	1.32 (0.81–2.14)	-
Yes	32		
Protestant			
No	46	0.96 (0.59–1.57)	-
Yes	29		
Muslim			
No	75	-	-
Yes	0		
Other			
No	61	0.76 (0.41–1.39)	-
Yes	14		
Education			
Low	17	1.44 (0.82–2.44)	-
High	56		
Income			
Low	35	0.80 (0.44–1.46)	-
High	17		
Blood transfusion			
No	69	1.06(0.44–2.55)	-
Yes	6		
*Sexual risk factors*			
Condom use			
High	16	0.76 (0.42–1.37)	-
Low	54		
Lifetime sexual partners			
< 10	65	0.93(0.46–1.88)	
≥ 10	10		
Age at first sex			
> 18	30	0.94 (0.58–1.54)	-
≤ 18	45		

*CI* confidence interval; *OR* odd ratio

Please note that all reference categories are listed as the first category

Although the total sample is 75, one data point is missing from condom use and two data points are missing from education level and income level. Four data points were omitted from condom use because the participant had zero sexual partners, 21 data points were omitted from income level because the participant was a student or housewife

*indicates a *p*-value <0.05, **indicates a *p*-value <0.01

In multivariate logistic regression analysis, after adjustment, HIV status continued to be associated with age (AOR = 2.35, 95 % CI = 1.24–4.46), education (AOR = 0.39, 95 % CI = 0.22–0.69), the number of lifetime sexual partners, (AOR = 2.26, 95 % CI = 1.22–4.17) and age at first sexual intercourse (AOR = 2.63, 95 % CI = 1.44–4.81) (Table 2). On the other hand, HBV was associated with sex, men being twice as likely to be infected compared to women (AOR = 0.46, 95 % CI = 0.28–0.75), and marital status, with those who were never married significantly more likely to be infected than those who were ever married (AOR = 1.66, 95 % CI = 1.01–2.73) (Table 3).

## Discussion

Our study hypothesized that there would not be an association between HBV and HIV, likely due to differing routes of transmission for the two diseases in Cameroon. We expected HIV status, but not HBV status to be associated with the high-risk sexual behaviour variables since we thought the main route of HBV transmission to be nonsexual. The results supported our hypothesis demonstrating that individuals infected with HBV were not significantly more likely to be infected with HIV compared to those without HBV. Furthermore, evidence supports the idea that HIV is spread through sexual transmission while it is unlikely that sexual intercourse is the main route of transmission for HBV. We found HIV status to be associated with high-risk sexual behaviour variables but did not find these variables to be associated with HBV. After adjustment, the number of sexual partners and age at first sex were associated with HIV status but lacked a significant association with HBV status. This may provide evidence that sexual intercourse was the main route of transmission for HIV in this population, but HBV may be acquired through routes other than sexual transmission.

There were many limitations to this study. Firstly, convenience samples from voluntary screenings are subject to selection bias. This may be one reason why we found a higher prevalence of HIV in our study population compared to published national statistics. Furthermore, the hepatitis B surface antigen will only test for individuals with chronic hepatitis B infection and therefore we have no information on those who had acute infection as a child and cleared the virus before adulthood. However, these individuals are of less concern since the virus is not causing ongoing harm to their livers. Since only 1 % of the study sample had ever been tested for HBV previously and our IRB protocol was only approved for individuals over the age of 18, it was impossible for us to gain information on the age of HBV transmission for this study. We were also confined to questions in our survey regarding sexual risk factors, and there may be more behaviours that could shed light on the relationship between these diseases and sexual practices. Additionally, counsellors at the health clinic administered questionnaires and individuals may have inaccurately reported sexual risk behaviours to please the counsellor or to avoid embarrassment. For example, of the 306 men participating in our study, none reported having homosexual intercourse. It is impossible to know whether our study only selected heterosexual males, whether the prevalence of homosexuality is extremely low in Cameroon, or whether participants felt uncomfortable sharing this information with the counsellor at the hospital. Further, our study relied on counsellors to record data on the questionnaires. Rarely, a counsellor failed to report one of the variables in the questionnaire, and this accounts for missing data. These errors were so infrequent that we do not believe they greatly affect the outcome of our study.

This study can be generalized to clinic populations in Southwest Cameroon and may be of interest to other sub-Saharan countries with similar HIV and HBV epidemics, especially in areas where there is a low rate of HBV immunization. Our sample population was fairly representative of the general population in Cameroon in terms of disease prevalence. The prevalence of HBV and HIV/HBV co-infection reflected similar findings as past studies [9, 10]. However, the prevalence of HIV is significantly higher in our study than the most recent data by UNICEF that found the prevalence of HIV to be 4.5 % among Cameroonian adults in a 2012. This may be due to the fact that our study took place in community hospitals and was open to anyone who wanted to participate. Therefore we attracted many patients attending the hospital for other reasons, including HIV treatment and counselling, and may have had a higher proportion of HIV-positive individuals compared to the public. In general, convenience-sampling models make it particularly difficult to obtain an accurate representation of the population as a whole.

Our study is one of few studies that goes a step beyond investigating a possible association between HIV and HBV and compares variables related to transmission routes of the two diseases. Future research should focus on factors specifically related to childhood transmission of HBV and reasons why the HBV transmission route may differ from other sexually transmitted diseases, when in fact HBV is easily spread through sexual intercourse. It is important to gain a better understanding of the specific risk factors contributing to the spread of disease such as sharing toothbrushes, human-to-human bites or contact between two open wounds. Understanding the most common routes of transmission is the first step in implementing prevention measures. Further evidence confirming that individuals are becoming infected in childhood and through nonsexual transmission may provide impetus to push the government to begin dispersing the HBV vaccine to infant vaccination centres and enact programs to educate individuals about transmission and testing practices.

## Conclusions

The prevalence of HIV and HBV was relatively high in this population, emphasizing the importance of intensifying activities of HIV prevention and treatment programs and implementing those for HBV in Cameroon. Additionally, the results from this study suggest that unlike HIV, HBV is not associated with sexual risk factors and may provide evidence that HBV is acquired through routes other than sexual transmission, warranting further investigation in this region.
